# Effect of Low-Frequency rTMS on Aphasia in Stroke Patients: A Meta-Analysis of Randomized Controlled Trials

**DOI:** 10.1371/journal.pone.0102557

**Published:** 2014-07-18

**Authors:** Cai-Li Ren, Guo-Fu Zhang, Nan Xia, Chun-Hui Jin, Xiu-Hua Zhang, Jian-Feng Hao, Hong-Bo Guan, Hong Tang, Jian-An Li, De-Liang Cai

**Affiliations:** 1 Department of Rehabilitation Medicine, Wuxi Tongren International Rehabilitation Hospital of Nanjing Medical University, Wuxi, Jiangsu Province, China; 2 Wuxi Mental Health Center of Nanjing Medical University, Wuxi, Jiangsu Province, China; 3 Department of Rehabilitation Medicine, The First Affiliated Hospital of Nanjing Medical University, Nanjing, China; University of Bologna, Italy

## Abstract

**Background:**

Small clinical trials have reported that low-frequency repetitive transcranial magnetic stimulation (rTMS) might improve language recovery in patients with aphasia after stroke. However, no systematic reviews or meta-analyses studies have investigated the effect of rTMS on aphasia. The objective of this study was to perform a meta-analysis of studies that explored the effects of low-frequency rTMS on aphasia in stroke patients.

**Methods:**

We searched PubMed, CENTRAL, Embase, CINAHL, ScienceDirect, and Journals@Ovid for randomized controlled trials published between January 1965 and October 2013 using the keywords “aphasia OR language disorders OR anomia OR linguistic disorders AND repetitive transcranial magnetic stimulation OR rTMS”. We used fixed- and random-effects models to estimate the standardized mean difference (SMD) and a 95% CI for the language outcomes.

**Results:**

Seven eligible studies involving 160 stroke patients were identified in this meta-analysis. A significant effect size of 1.26 was found for the language outcome severity of impairment (95% CI = 0.80 to 1.71) without heterogeneity (*I^2^* = 0%, *P* = 0.44). Further analyses demonstrated prominent effects for the naming subtest (SMD = 0.52, 95% CI = 0.18 to 0.87), repetition (SMD = 0.54, 95% CI = 0.16 to 0.92), writing (SMD = 0.70, 95% CI = 0.19 to 1.22), and comprehension (the Token test: SMD = 0.58, 95% CI = 0.07 to 1.09) without heterogeneity (*I^2^* = 0%). The SMD of AAT and BDAE comprehension subtests was 0.32 (95% CI = −0.08 to 0.72) with moderate heterogeneity (*I^2^* = 32%,*P* = 0.22). The effect size did not change significantly even when any one trial was eliminated. None of the patients from the 7 included articles reported adverse effects from rTMS.

**Conclusions:**

Low-frequency rTMS with a 90% resting motor threshold that targets the triangular part of the right inferior frontal gyrus (IFG) has a positive effect on language recovery in patients with aphasia following stroke. Further well-designed studies with larger populations are required to ascertain the long-term effects of rTMS in aphasia treatment.

## Introduction

Stroke is a primary cause of disability worldwide and contributes considerably to the global disease burden (WHO 2011). Approximately one-third of all stroke patients develop aphasia [1], [2]. These patients are more likely to have extended hospitalizations and use rehabilitation services more often than stroke patients without aphasia [3]. Evidence suggests that aphasia decreases quality of life, as one in three aphasia patients is diagnosed with depression 12 months post-stroke [4], [5]. Therefore, effective therapeutic strategies are needed to treat aphasia in stroke patients. Intensive speech and language therapy (SLT), one of the effective treatments for aphasia, has been demonstrated to improve outcomes for affected stroke patients [6]. However, a recent systematic review showed that there was insufficient evidence to support the effectiveness of any one specific SLT approach over another, although it did provide evidence of the effectiveness of SLT for people with aphasia following stroke in terms of improved functional communication, receptive language, and expressive language [7]. Additionally, a recent meta-analysis found no evidence that transcranial direct current stimulation (tDCS) enhanced SLT outcomes [8]. Thus, other effective adjunctive therapies with or without SLT should also be considered.

Repetitive transcranial magnetic stimulation (rTMS) is a noninvasive brain stimulation method that may improve stroke rehabilitation [9], [10]. rTMS may relatively normalize the neural activity in the cortical area of metabolic dysfunction and may elicit an excitatory or inhibitory effect on the neurons of the targeted brain area. High-frequency rTMS (>1 Hz) has been shown to transiently facilitate neural activity. An advantage of inhibitory, low-frequency rTMS (≤1 Hz) is that it regulates the level of excitability of a given cortical area beyond the duration of the rTMS train itself [11], [12]. The benefits of cortex modulation with 1 Hz rTMS in post-stroke aphasic patients have been demonstrated in preliminary studies involving individual cases or groups of chronic participants [13]–[15]. As an adjunct to therapies for aphasia, rTMS may further improve the effectiveness of SLT for aphasia after stroke. Randomized controlled pilot studies have demonstrated that 1 Hz rTMS applied to the right pars triangularis has the ability to improve language recovery and to modulate neural language networks [16]–[22]. The underlying mechanisms involved in the application of rTMS to a homologous language region may include neural reorganization mechanisms resulting in a prospective reduction in interhemispheric inhibition [23], [24]. However, no systematic review or meta-analysis study has been conducted to identify the effects of all available trials. Thus, a meta-analysis of all randomized controlled trials (RCTs) was conducted to examine the effectiveness and acceptability of rTMS. The goal of the present study was to evaluate the effects of rTMS on aphasia in stroke patients relative to sham rTMS. The secondary aim was to examine possible adverse effects of using rTMS in stroke patients.

## Methods

### Search strategy

Electronic searches were performed in the PubMed, Cochrane Central Register of Controlled Trials (CENTRAL), Embase, CINAHL, ScienceDirect, and Journals@Ovid databases to identify relevant studies. The search terms were “aphasia OR language disorders OR anomia OR linguistic disorders AND repetitive transcranial magnetic stimulation OR rTMS.” The searches were limited to human studies that were written in English and published between January 1965 and October 2013. We did not register a protocol of the present meta-analysis on the Cochrane library.

### Study selection

The following inclusion criteria were applied: (1) the patients were diagnosed with stroke, (2) the patients were adults, (3) ≥5 participants were recruited, (4) the focus was on the effects of rTMS on aphasic patients after stroke, (5) the outcome measures were reported with continuous scales that evaluated the degree of language impairment, and (6) the study was a randomized controlled trial. Two reviewers (CLR and GFZ) independently searched and evaluated the literature for the inclusion of studies based on their titles and abstracts. If the summary appeared to be relevant, the complete text was obtained to evaluate its methodological quality.

### Qualitative analyses

Eligible studies were assessed for risk of bias using the Cochrane Collaboration tool. The Cochrane tool classifies studies as having a low, high, or unclear risk of bias across seven domains: sequence generation, allocation concealment, blinding (self-report outcomes), blinding (objective outcomes), missing data, selective reporting, and other biases.

### Data extraction

Data from each study were independently extracted by two authors (CLR and GFZ) using a standard data-recording form that included the study design, number of subjects, mean age, stroke duration, treatment protocol (i.e., rTMS frequency, intensity, number of pulses, and additional interventions), dropout number, information regarding study quality, outcome measures, and pretreatment and post-treatment means and standard deviations for outcome measure (where reported). Various aphasia assessment outcome measures were used across the studies, some of which assessed multiple measures. For the purposes of this meta-analysis, the measure used to assess each study was the explicitly declared primary outcome. If the primary outcome was not clearly defined, the first outcome reported with a mean and SD in the results section was used. Disagreements between the authors on the eligibility of studies were discussed with a third author to reach consensus.

### Data synthesis

The standardized mean difference was used to include these data in a meta-analysis in which a single outcome measure was assessed and reported across trials using different measurement tools; the mean difference was applied using the same measurement tool. In cases in which the direction of measurement differed, it was necessary to adjust the direction of certain measures to ensure that all scales operated in the same direction. For example, measures of naming ability generally increase with increasing ability; however, in some cases, improving naming skills might be reflected by decreased scores. Therefore, it was necessary to multiply the mean values by −1 to ensure that all scales performed in the same direction. Standard deviation (SD) values were unaffected, and we have presented these within the meta-analysis without the need for a directional change.

Statistical analyses were conducted using RevMan 5.2 software from the Cochrane Collaboration. The standardized effect sizes and 95% CIs were calculated to test the results of the different trials. Absolute effect sizes that ranged from 0.2 to 0.49 were considered to be minimally important differences, and a value of 0.5 was considered clinically significant [25].

### Assessment of heterogeneity

The heterogeneity across each effect size was evaluated with Q-statistics and the *I^2^* index, which is useful for assessing consistency between trials [26]. A random-effects model was used when significant heterogeneity was observed by Q-statistics or when *I^2^* was >50%. When *I^2^* was <50%, a fixed-effects model was applied. Sensitivity analysis was conducted to determine the robustness of the pooled results. A funnel plot, rank correlation and a regression test were used to describe possible publication bias.

## Results

### Characteristics of the included studies

**Figure 1:**
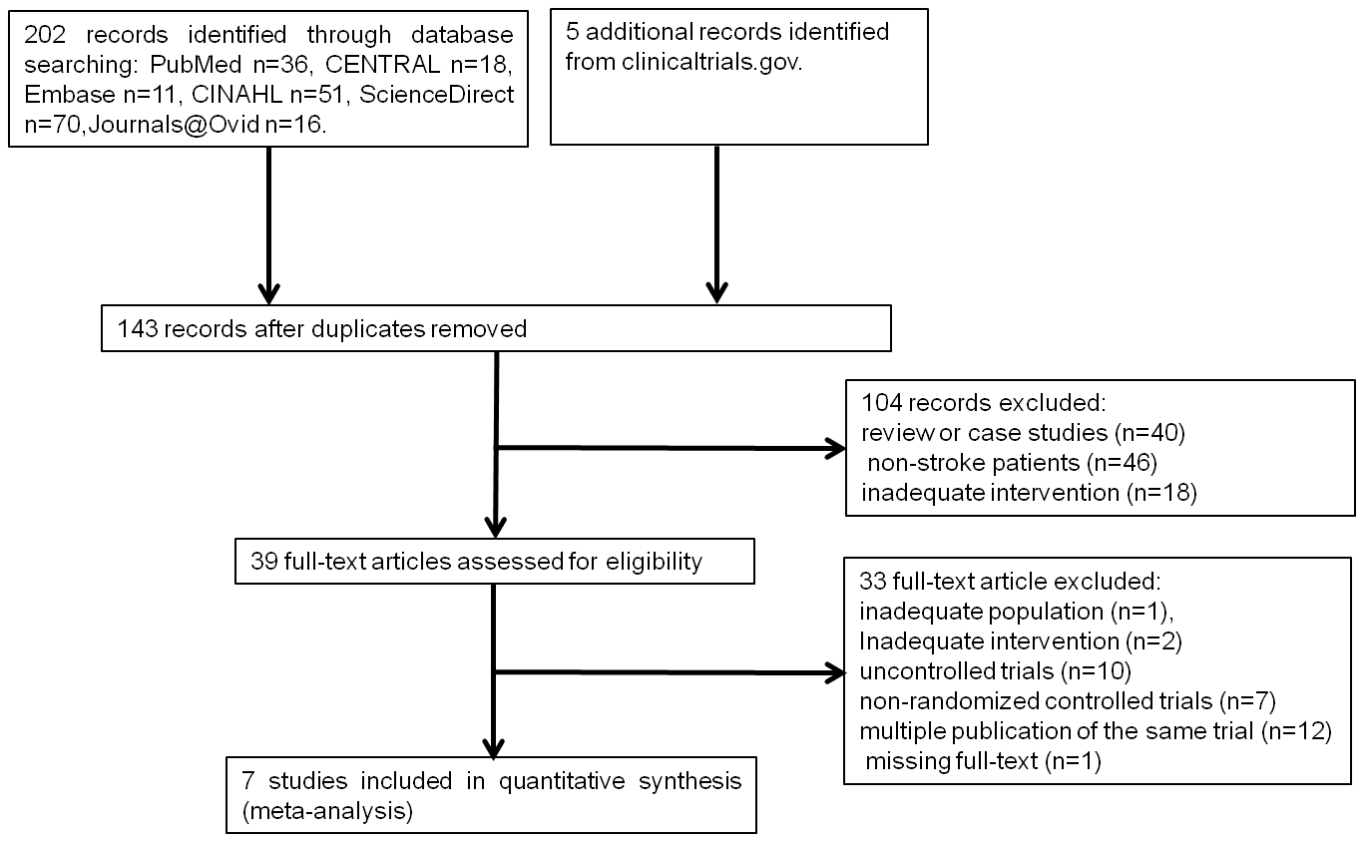
Flowchart for the inclusion of studies.

We identified 202 unique records from the database searches. After screening the titles and abstracts, we excluded records and obtained the full texts of the remaining 39 articles. After further assessment, seven studies fulfilled the inclusion criteria. We also identified five trials from clinicaltrials.gov. Three studies have been completed but have not been published, and two trials are ongoing, but we have been unable to obtain unpublished data. Seven studies involving a total of 160 participants were included. All studies investigated the effect of rTMS versus sham rTMS. Six trials explored the effect of rTMS combined with speech and language therapy (SLT) [16]–[19], [21], [22]. We excluded seven trials about the effect of rTMS on poststroke aphasia, primarily because they were not RCTs [14], [27]–[32]. The flow of references is illustrated in Figure 1.

Table 1 shows the main characteristics of the included studies in our meta-analysis. A total of 160 participants were randomized across seven randomized comparisons that contrasted real rTMS with sham rTMS. The mean patient age reported in the seven trials ranged from 59.7 to 71.2 years. All participants suffered from ischemic infarct within the left middle cerebral artery territory. All patients were right-handed. Six trials indicated the length of time elapsed since the participants had experienced the onset of their aphasia; the widest time range post-onset was 26 to 75 months [16]. The shortest mean length of time since the onset of the participants' aphasia was 33.5 days (range, 9.4 to 68.7 days) [17]. Using the 6-point Aphasia Severity Rating Scale (ASRS), the severity of aphasia was reported by 3 trials [17], [20], [21]. The therapeutic procedures used in six trials consisted of rTMS sessions and specific language training. In these trials, immediately after finishing the rTMS treatment, both the experimental and control participants underwent speech and language therapy sessions for 45 minutes. The patients of the other study were only treated with real rTMS or sham rTMS sessions [20]. In all trials, rTMS was performed with a Magstim Rapid stimulator (Magstim Company, Whitland, UK) equipped with an air-cooled figure-of-eight coil (each loop measured 70 mm in diameter). All trials used 1 Hz rTMS with an intensity equaling 90% of the daily defined individual resting motor threshold. The treatment and sham stimulation sessions of five trials [16], [18]–[20], [22] were conducted 5 days per week for a 2-week period, whereas those of two trials [17], [21] were performed for a 3-week period. All included trials targeted the triangular part of the right inferior frontal gyrus (IFG). The sham stimulation condition of three studies [17], [20], [21] was performed with an air-cooled sham coil that looks and sounds similar to the discharge of real TMS coil. The sham coil was placed at the same site on the scalp and with the same stimulation parameters used for the real rTMS procedure. The other four studies [16], [18], [19], [22] used the same coil used the real rTMS placed over the vertex. All trials measured language outcomes. In those cases in which the data for this comparison were available, they are presented below in relation to the following parameters: (1) severity of impairment, (2) expressive language, and (3) receptive language.

**Table 1:** Characteristics of the Included Studies.

Study	No. of Participants (Exp/Ctr)	Mean Age, Y (Exp/Ctr)	Stroke Duration	First Language	rTMS Protocol	Coil/Position	Sham rTMS Method	Main Outcome Measures for Motor Function
**Barwood 2013**	12(6/6)	(60.8/67)	26–75 m	English	1 Hz, 90% MT, 1200 pulses, 20 min, for 10 days (10 sessions)	Figure 8/the triangular part of the right posterior IFG	Sham coil	BNT, BDAE, picture naming inventory
**Thiel 2013**	24(13/11)	(69.8/71.2)	19.0–73.23 d	German	1 Hz, 90% MT, 1200 pulses, 20 min, for 10 days (10 sessions)	Figure 8/the triangular part of the right posterior IFG	Stimulation over vertex	AAT
**Seniow 2013**	38(19/19)	(61.8/59.7)	9.4–68.7 d	Polish	1 Hz, 90% MT, 1800 pulses, 30 min, for 15 days (15 sessions)	Figure 8/the triangular part of the right IFG	Sham coil	BDAE
**Hartmann 2013**	21(11/10)	/	/	German	1 Hz, 90% MT, 20 min, for 10 days	Figure 8/the triangular part of the right IFG	Stimulation over vertex	AAT
**Heiss 2013**	29(15/14)	(68.5/69)	21.27–74.06 d	German	1 Hz, 90% MT, 20 min, for 10 days	Figure 8/the triangular part of the right IFG	Stimulation over vertex	AAT
**Waldowski 2012**	26(13/13)	(62.31/60.15)	9.53–80.87 d	Polish	1 Hz, 90% RMT, 1800 pulse, 30 min, for 15 days	Figure 8/the triangular part of the right IFG	Sham coil	CPNT
**Weiduschat 2011**	10(6/4)	(66.6/63.75)	18–97 d	German	1 Hz, 90% RMT, 20 min, for 10 days	Figure 8/the triangular part of the right IFG	Stimulation over vertex	AAT subtest and total score

BNT: Boston Naming Test, BDAE: Boston Diagnostic Aphasia Examination, AAT: Aachen Aphasia Test., CPNT: Computerized Picture Naming Test.

### Risk of bias in the included studies

**Figure 2:**
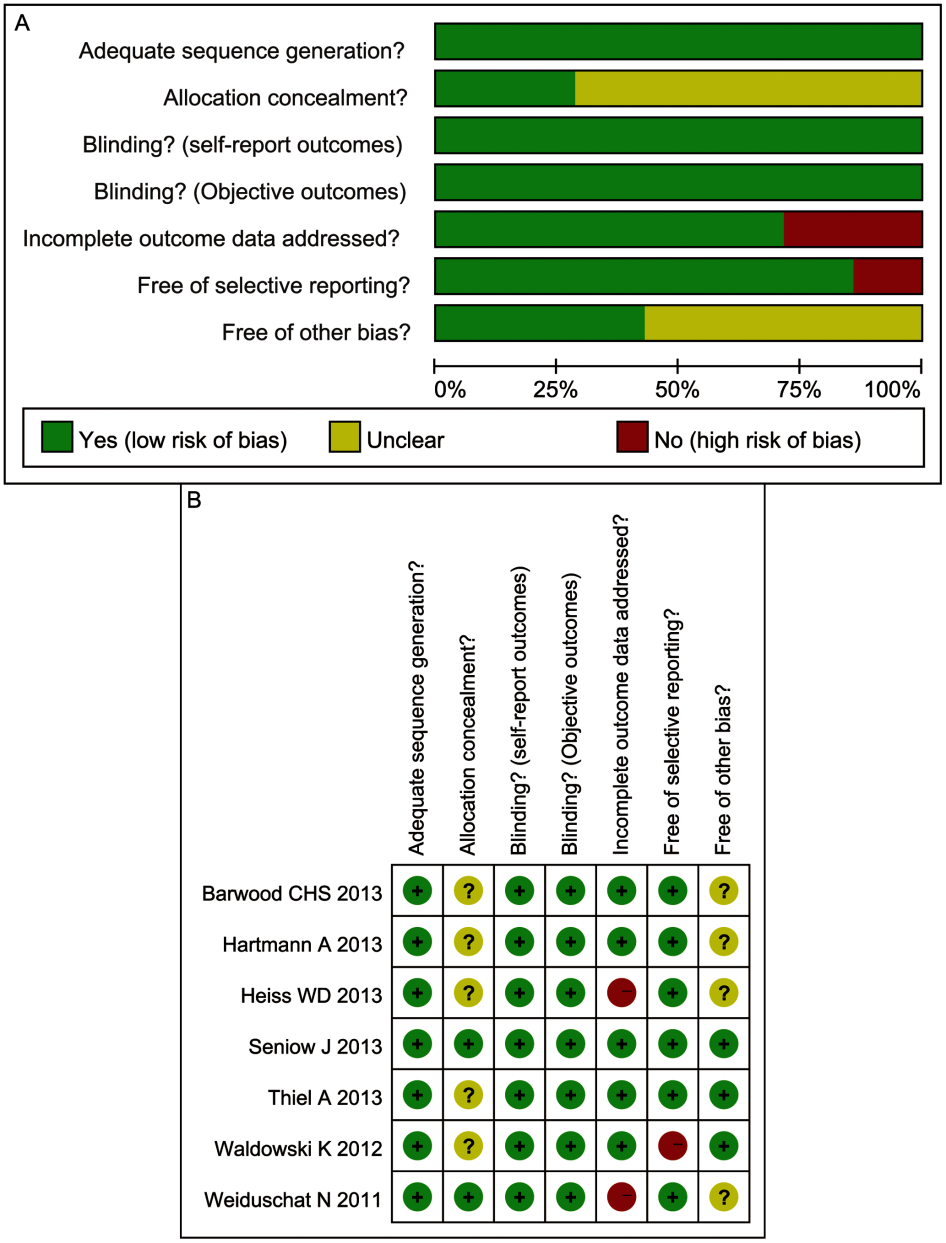
Risk of bias graph (A): overview of the authors' judgments about each risk of bias item presented as percentages across all included studies. Risk of bias summary (B): overview of the authors' judgments about each risk of bias item for each included study.

The risk of bias tool, implemented in RevMan 5.2, was used to assess the risk of bias according to the conditions described in the Methods section. Information on the risk of bias at the study level is shown in Figure 2(A and B). All seven included studies (100%) exhibited a low risk of bias for sequence generation [16]–[22], and three of them [17], [20], [21] explicitly described the randomization procedures. The other four studies did not provide the specific methods used. Two studies (29%) exhibited a low risk of bias for concealment of allocation by using random number generators and sealed, numbered envelopes [17], [22]. All of the included studies exhibited a low risk of bias for blinding the participants, personnel and the outcomes assessment [16]–[22]. Two studies were at a high risk of bias for incomplete outcomes [18], [22], whereas the remaining five studies [19]–[21] (70%) showed a low risk of bias in this category. Six of the seven included studies [16]–[20], [22] (86%) exhibited a low risk of bias for selective outcome reporting, and one study [21] exhibited a high risk of bias. Three [16], [17], [21] of the seven included studies (43%) exhibited a low risk of bias for other biases, and the risk of bias of the remaining four studies (57%) [18]–[20], [22] was unclear.

### Primary outcomes

#### Severity of aphasia impairment

**Figure 3:**
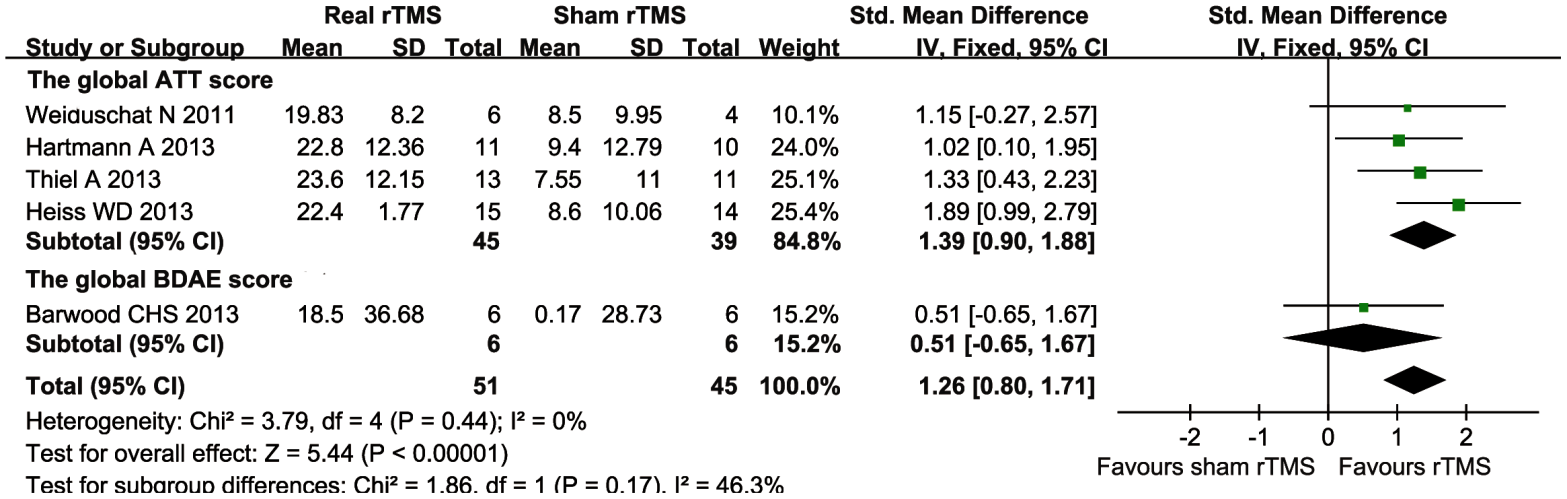
Forest plot of SMD and 95% CI for the severity of language impairment in patients received rTMS and sham rTMS.

Five trials [16]–[20], [22] compared the active rTMS group with a group that received sham rTMS by measuring the severity of each participant's aphasia impairment. The language assessment batteries included the Aachen Aphasia Test (AAT) global scores [16], [18], [19], [22] and the Boston Diagnostic Aphasia Examination (BDAE) [17], [20]. We obtained statistical summary data suitable for inclusion within a meta-analysis from these five trials. Pooling the available data using SMDs, we observed no heterogeneity (*I^2^* = 0%, *P* = 0.44). The data were pooled using a fixed-effects model. There was a significant difference between the real rTMS groups and sham rTMS groups (SMD = 1.26, 95% CI = 0.80 to 1.71, *P*<0.01) (Figure 3). Sensitivity analyses were conducted after omitting Heiss WD's study, which has an unclear risk of allocation concealment bias and a high risk of incomplete outcome bias (SMD = 1.04, 95% CI = 0.52 to 1.56, *P*<0.01). The meta-analysis continued to show that there was a statistically significant effect of rTMS compared with sham rTMS on the severity of aphasia.

#### Expressive language

**Figure 4:**
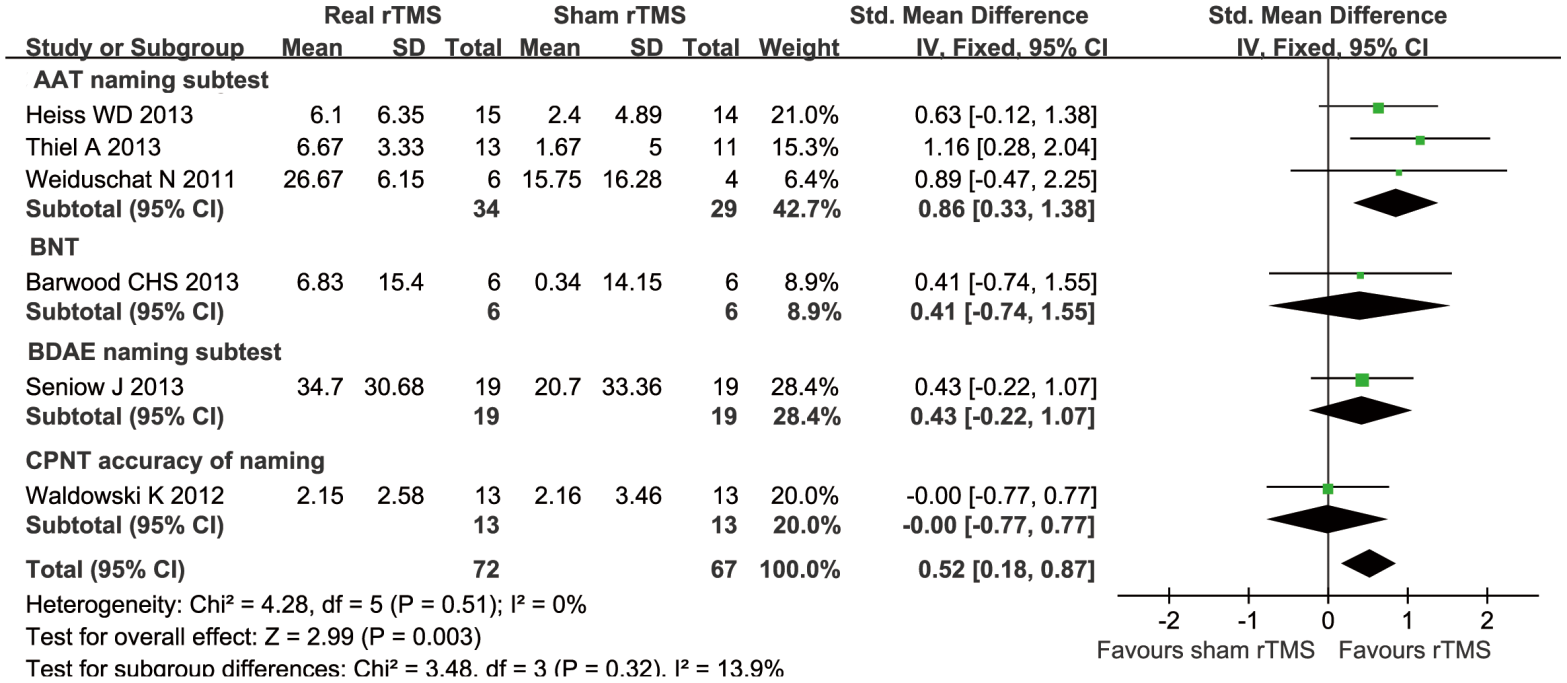
Forest plot of SMD and 95% CI for the outcome of naming in patients received rTMS and sham rTMS.

**Figure 5:**
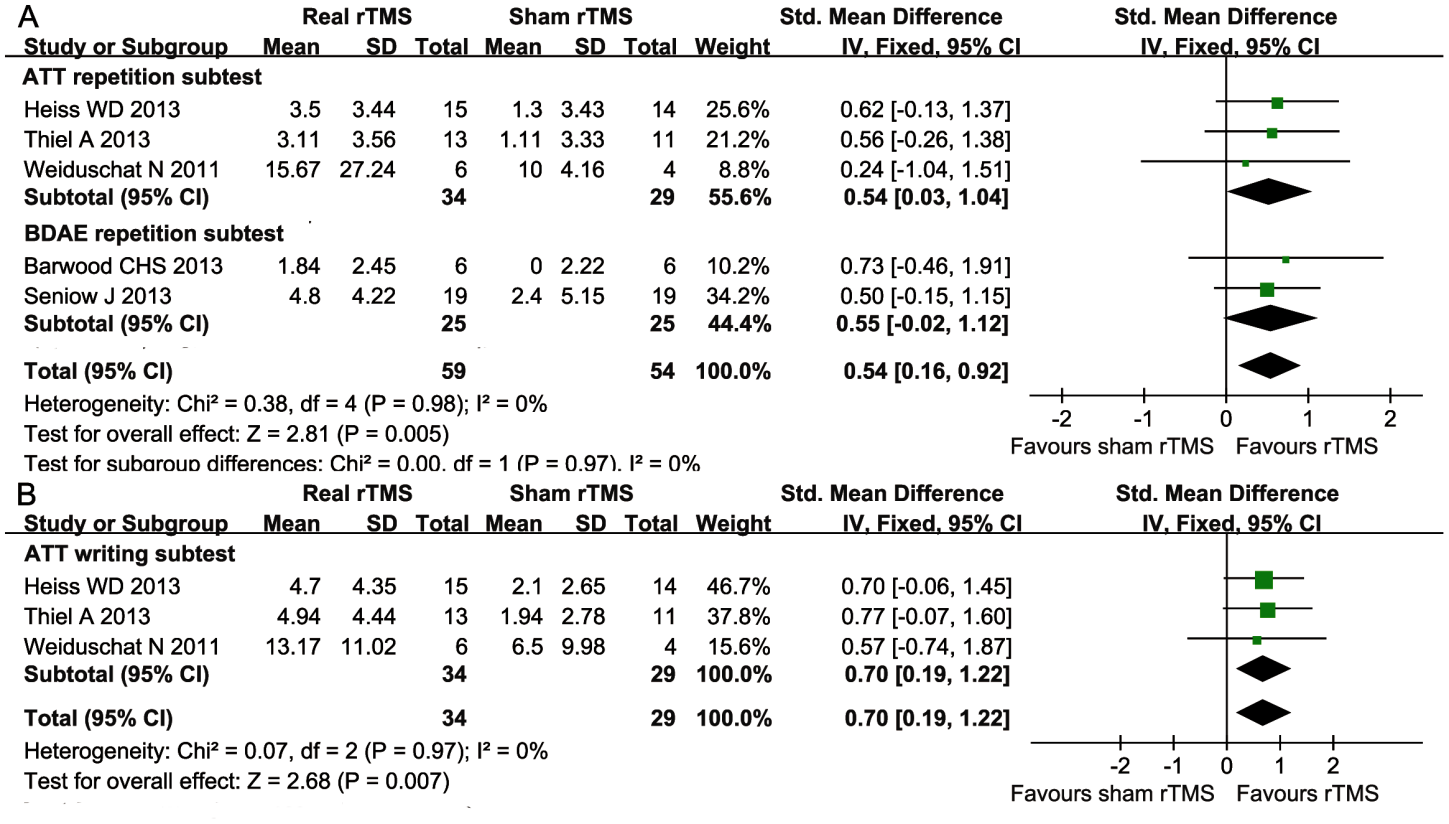
Forest plot of SMD and 95% CI for the outcome of repetition (A) and writing (B) in patients received rTMS and sham rTMS.

Six trials [16]–[18], [20]–[22] formally evaluated the participants' expressive language skills using naming (i.e., the Boston Naming Test [BNT], BDAE naming subtests, AAT naming subtests, and specially designed Computerized Picture Naming Test [CPNT]) and repetition (the BDAE and AAT repetition subtests). Written language expressive skills were measured using the AAT writing subtests and the BDAE writing subtests. The meta-analysis of naming, repetition, and writing showed a statistically SMD of 0.52 (95% CI = 0.18 to 0.87; *P* = 0.003), 0.54 (95% CI = 0.16 to 0.92, *P* = 0.0009), and 0.70 (95% CI = 0.19 to 1.22, *P* = 0.007), respectively, without heterogeneity (*I^2^* = 0%, *P* = 0.51) (*I^2^* = 0%, *P* = 0.98) (*I^2^* = 0%, *P* = 0.97) (Figure 4 and 5). We observed that a clinically significant effect size (SMD>0.50) was obtained when one trial was eliminated.

#### Receptive language

**Figure 6:**
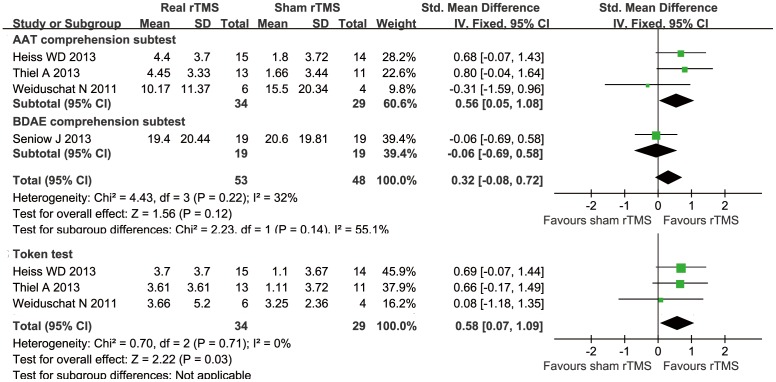
Forest plot of SMD and 95% CI for the outcome of comprehension in patients received rTMS and sham rTMS.

Four of the seven trials measured the participants' receptive language comprehension skills [16]–[18], [22], and auditory comprehension was measured using the Token Test and AAT and BDAE subtests. After pooling the Token Test data, the meta-analysis indicated an SMD of 0.58 (95% CI = 0.07 to 1.09; *P = 0.03*) with no heterogeneity (*I^2^* = 0%, *P* = 0.71), while the meta-analysis of the AAT comprehension subtest and the BDAE comprehension subtest indicated an SMD of 0.32 (5% CI = −0.08 to 0.72; *P = 0.12*) with moderate heterogeneity (*I^2^* = 32%, *P* = 0.22) (Figure 6).

### Secondary outcomes

#### Adverse effects

None of the seven trials reported any adverse effects.

### Analysis for publication bias

**Figure 7:**
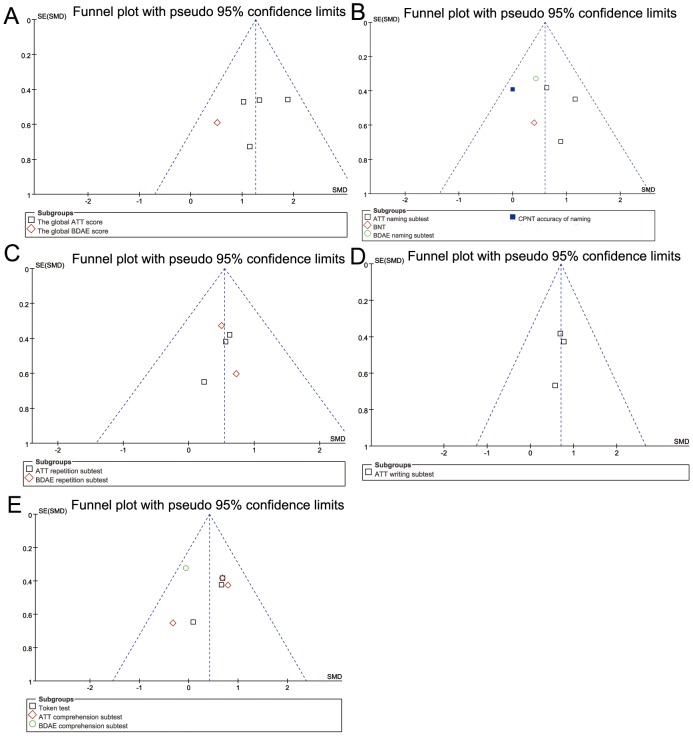
Funnel plots demonstrating publication bias for the severity of impairment (A), naming (B), repetition (C), writing (D), and comprehension (E).

As shown in the funnel plots in Figure 7, no publication bias was observed for severity of aphasia, naming, repetition, writing or comprehension (Egger's test: *P* = 0.758, *P* = 0.379, *P* = 0.902, *P* = 0.545, *P* = 0.768 respectively, and Begg's test: *P* = 0.308, *P* = 0.348, *P* = 1.000, *P* = 1.000, *P* = 1.000, respectively).

## Discussion

### Summary of the main results

The present study supports the efficacy of using low-frequency rTMS in the right homologs of Broca's area on language recovery in aphasia patients with stroke. No statistical evidence was found for publication bias or heterogeneity, and the results remained significant after any one of the trials was removed.

The results of this meta-analysis suggest that significant differences between the groups' scores were evident in measures of language impairment, receptive language, and expressive language, all of which favored the use of low-frequency rTMS. In the present meta-analysis, the significant mean effect size was 1.26 for severity of aphasia, 0.52 for naming, 0.54 for repetition, 0.70 for writing and 0.58 for the Token Test, which all indicate clinically significance. This result is also supported by some case reports and open-protocol studies, which have indicated that significant improvements were obtained in naming or picture naming after applying 1 Hz rTMS over the right homologue of Broca's area [31]. An SMD of only 0.32 was obtained for the AAT and BDAE comprehension tests, which indicated that there was no statistically significant effect of real rTMS compared with sham rTMS on the outcome of those tests. Martin et al. showed that not all aphasic patients responded well, and that lesion site may play a role in each patient's response to TMS treatment [32]. Some enrolled clinical trials have established the underlying mechanism by which the application of rTMS to a homologous language region induces neural reorganization and reduces interhemispheric competition [16], [18], [22]. Consistent with these observations, Hsu et al. showed that rTMS over the unaffected hemisphere has a positive effect on motor recovery by balancing of interhemispheric competition [33].

The follow-up times differed in each trial, and only three trials reported the effect of rTMS follow-up times after treatment, which complicated further data analysis. Two trials followed up with patients 15 weeks after treatment, and one followed up with patients 2, 8, and 12 months after treatment. One 15-week follow-up study revealed that severely aphasic rTMS patients demonstrated significantly greater improvements than those receiving repeated sham stimulation [17]. Another study showed that the rTMS subgroup with lesions that included the anterior part of the language area showed greater improvement primarily in naming reaction time 15 weeks after treatment [21]. Similar observations were reported by Barwood et al., who observed improved accuracy in naming on a number of subtests of the BDAE and Snodgrass & Vanderwart (1980) naming inventory up to 12 months after stimulation. These results suggest the long-term effects of follow-up on naming and repetition after rTMS treatment. Multicenter studies with large patient samples are needed to investigate the long-term effect of rTMS on aphasia.

The present meta-analysis is limited to low-frequency rTMS protocols and does not include other protocols, such as high-frequency rTMS or patterned rTMS, theta burst stimulation (TBS). Some studies have showed that TBS over the right Broca's homologue improves naming performance in aphasic patients [28], [34]. These studies were excluded as crossover trials or case reports. Still other studies confirmed that high-frequency rTMS over the left dorsolateral prefrontal cortex (DLPFC) decreases vocal reaction times for picture naming in healthy individuals [35], [36], increases the number of correct responses in patients with Alzheimer's disease [37], [38], and facilitates action-naming performance in patients with progressive non-fluent aphasia (PNFA) [39]. However, the effect of high-frequency rTMS in stroke aphasia patients has not yet been studied in a randomized clinical trial.

Safety is an important consideration because rTMS can produce potential adverse effects, such as headaches and seizures. Thus, we investigated adverse effects in the present meta-analysis. No severe adverse effects were reported in the included studies. None of the patients reported that their language impairment worsened after treatment. This study suggests that rTMS is a safe treatment in the short term, but long-term follow-up is needed to further investigate the safety of this treatment. Although rTMS is generally assumed to be safe in patients following stroke, investigators should follow safety guidelines and examine the potential risk of post-stroke seizure related to rTMS.

### Overall completeness and applicability of evidence

The results of this meta-analysis can be generalized for following conditions: (1) most patients are first-time stroke patients, (2) the majority of participants suffer from ischemic stroke, (3) nearly all participants are right-handed, and (4) 1 Hz rTMS with 90% RMT, targeting the triangular part of the right inferior frontal gyrus, is performed. Hence, the results may be of limited applicability for individuals with recurrent and hemorrhagic strokes and for left-handed patients. This meta-analysis also failed to subgroup the results by aphasic severity degree and aphasic syndrome. The current meta-analysis provides sufficient evidence to draw conclusions about the benefits of low-frequency rTMS in stroke aphasia.

### Quality of the evidence

The seven included trials were randomized, prospective, placebo-controlled studies, of which six clearly described the double-blinding method. One trial [19] stated that double-blinding was used but did not clearly describe who was blinded. Two trials [17], [22] clearly described allocation concealment. In five trials, allocation concealment was mentioned but the procedures were unclear. In all seven trials, incomplete outcome data were addressed adequately. The drop-out rates in two studies were low (5% and 20% in [17] and [16] respectively), and three studies had no drop-outs [19]–[21]. The risk of incomplete outcome data bias for these five studies was therefore moderately low. The Weiduschat 2011 [18] and Heiss 2013 [22] trials had a 28.5% and 29% drop-out rate, respectively, creating a potentially high risk of incomplete outcome data bias. Six trials [16]–[20], [22] reported all pre-specified, expected results. The other study [21] did not report all of the results, indicating a risk of selective reporting bias.

Our study has several limitations: (1) we did not include any unpublished works; (2) only seven studies were included, which made subgroup meta-analysis according to stroke phase or aphasia type difficult; (3) publication bias might have affected our results. Although the funnel plot for our main outcome did not show evidence of publication bias, as measured by visual inspection, this result does not mean that no publication bias existed. Finally, we may have overlooked relevant studies that were published in languages other than English.

## Conclusions

This meta-analysis indicates a clinically positive effect of rTMS with or without SLT for patients with aphasia following stroke in overall language function and expressive language, including naming, repetition, writing, and comprehension. Low-frequency (1 Hz) rTMS over the unaffected hemisphere is effective and compatible with the concept of interhemispheric inhibition. Moreover, the treatment of 1 Hz rTMS for patients with aphasia after stroke was safe. No adverse effects were observed in patients in all seven trials. However, further well-designed studies are necessary to determine the effect duration and long-term impact.

## Supporting Information

Checklist S1 **The PRISMA checklist for the manuscript.**
(DOC)
